# Analysis of Fluid Replacement in Two Fluidic Chambers for Oblique–Incidence Reflectivity Difference (OI-RD) Biosensor

**DOI:** 10.3390/s24062000

**Published:** 2024-03-21

**Authors:** Haofeng Li, Mengjing Xu, Xiaohan Mai, Hang Zhang, Xiangdong Zhu, Lan Mi, Jiong Ma, Yiyan Fei

**Affiliations:** 1 Department of Optical Science and Engineering, Shanghai Engineering Research Center of Ultra-Precision Optical Manufacturing, Key Laboratory of Micro and Nano Photonic Structures (Ministry of Education), School of Information Science and Technology, Fudan University, Shanghai 200433, China; 21210720003@m.fudan.edu.cn (H.L.); 21110720127@m.fudan.edu.cn (M.X.); 22210720012@m.fudan.edu.cn (X.M.); hangzhang20@fudan.edu.cn (H.Z.); lanmi@fudan.edu.cn (L.M.); jiongma@fudan.edu.cn (J.M.); 2 Department of Physics, University of California, One Shields Avenue, Davis, CA 95616, USA; xdzhu@physics.ucdavis.edu

**Keywords:** optical biosensor, fluidic chamber, COMSOL, simulation, fluid replacement

## Abstract

Optical biosensors have a significant impact on various aspects of our lives. In many applications of optical biosensors, fluidic chambers play a crucial role in facilitating controlled fluid delivery. It is essential to achieve complete liquid replacement in order to obtain accurate and reliable results. However, the configurations of fluidic chambers vary across different optical biosensors, resulting in diverse fluidic volumes and flow rates, and there are no standardized guidelines for liquid replacement. In this paper, we utilize COMSOL Multiphysics, a finite element analysis software, to investigate the optimal fluid volume required for two types of fluidic chambers in the context of the oblique–incidence reflectivity difference (OI-RD) biosensor. We found that the depth of the fluidic chamber is the most crucial factor influencing the required liquid volume, with the volume being a quadratic function of the depth. Additionally, the required fluid volume is also influenced by the positions on the substrate surface bearing samples, while the flow rate has no impact on the fluid volume.

## 1. Introduction

Optical biosensors have a significant impact on various aspects of our lives and find extensive applications in biotechnology, pharmacy, medical diagnosis, the food industry, and environmental engineering [1,2]. Fluidic chambers play a crucial role in optical biosensors by facilitating the controlled delivery of fluids in various applications such as drug screenings [3], the assessment of reaction kinetics of molecules [4], detection of pathogens [5], and measurement of protein adsorption [6,7]. Fluidic chambers find applications in various optical biosensors, including surface plasmon resonance (SPR) [8,9] and ellipsometry [10,11,12], enabling in situ and real-time measurements. Different configurations of fluidic chambers are utilized in these biosensors for different biosensing approaches and various application scenarios. It is crucial to recognize that fluidic parameters can significantly differ among different fluidic chambers.

Microfluidic technology is widely used in biosensors to create small fluidic chambers due to its cost-effectiveness and capacity for high throughput [9,13]. These fluidic chambers play an important role in facilitating the transportation and replacement of sample and buffer solutions, allowing for the interaction between immobilized biomolecules and analytes in an aqueous solution [14]. For instance, Tokel et al. [15] designed a microfluidic chip for pathogen detection that consisted of five layers: PMMA (poly methyl methacrylate), DSA (an adhesive), PMMA, DSA, and gold. The DSA layer contained a 50 μm-thick channel that served as the fluidic chamber, forming a flow cell with the gold film and the PMMA layer. The volume of this fluidic chamber was 4 μL. To ensure reliable and robust conditions, a solution of 100 μL was flowed through the chip at a flow rate of 5 μL/min during the detection process. Gaspar et al. [16] introduced a novel microfluidic chip design that incorporated an ultra-low volume fluidic chamber. This chip comprised two permanently bonded PDMS (polydimethylsiloxane) layers, each featuring a hole aligned concentrically through which a capillary tube was inserted. When pressed against a gold film, the structure formed a reversible bond. Unlike traditional flow channels, this specially designed fluidic chamber utilized the narrow gap between the tube end and the gold film to create a fluidic chamber with a volume of less than 1 nL. The recommended flow rate for this fluidic chamber was 3 μL/min, and a fluidic volume of 20 μL was recommended to ensure reliable and accurate results. Wang et al. [17] developed a microcell array made of PDMS to enhance throughput and efficiency for immunoassays. The array consisted of 48 small fluidic chambers, each with a depth of 50 μm and a volume of 18 nL. The system operated at a typical flow rate of 1 μL/min, requiring a fluidic volume of 10 μL for complete liquid replacement.

Apart from biosensors used in laboratories, many commercial biosensors also utilize microfluidic chips as fluidic chambers. One such example is the Biacore instrument, which is widely used for measuring the affinity between two biomolecules through SPR biosensing. In the Biacore system, an integrated μ-fluidic cartridge (IFC) is used to deliver the sample solution over the gold film of the sensor chip, where the biomolecules are immobilized. When the sensor chip docks onto the IFC, the grooves on the IFC and the gold film create fluidic chambers [18]. The Model Biacore 2000 contains four fluidic chambers, each with a volume of 0.02 μL [19], while the Model Biacore X100 has two fluidic chambers, with each chamber having a volume of 0.06 μL. To immobilize molecules on the sensor chip, it is recommended to use a moderate flow rate (5–20 μL/min, usually 10 μL/min). However, for kinetic experiments, it is recommended to use a higher flow rate exceeding 30 μL/min (usually 50 μL/min) to minimize sample depletion [20]. During experiments, dozens or hundreds of microliters of sample solution are injected into the fluidic chambers to ensure reliable results.

Although microfluidic technology has greatly reduced sample consumption by creating small fluidic chambers, there are still situations where larger fluidic chambers are needed for specific applications. Karlsson et al. [21] proposed a lengthy fluidic chamber, with a cross-section in the shape of an isosceles trapezoid. The sample is positioned on the backside of this chamber, and the non-parallel sides, made of quartz, are perpendicular to the laser beam. The volume of this chamber is approximately 50 mL. The flow rate is typically 3 L/min, and a fluidic volume of 500 mL is used for complete liquid replacement. Byrne et al. [7] developed a tubular fluidic chamber, which consists of a 146 mm-long fused quartz pipe with an aluminum semi-rod positioned inside. The samples are adsorbed on the centerline of the semi-rod, which overlaps with the axis of the pipe, allowing the incoming and outgoing laser beams to pass through perpendicularly. The total volume of this fluidic chamber is 3.7 mL, and solutions are pumped into it at a rate of 2.5 mL/min. Usually, a fluidic volume of 150 mL is sufficient for a protein layer to form on the surface of the semi-rod.

Evidently, significant variations exist in parameters, such as flow rate and solution volume, among different fluidic chambers in optical biosensors. Specifically, to ensure complete liquid replacement, the required volume of replacement liquid can vary from 10 times to 2000 times the volume of the fluidic chamber [7,15,16,17,19,20,21]. This wide range suggests the absence of standardized guidelines for liquid replacement volume. Furthermore, using a replacement liquid volume that is excessively larger than the volume of the fluidic chamber results in excessive sample consumption. In the case of precious protein samples with limited quantities, there may not be a sufficient volume available for replacement. Therefore, it is desirable to flow a minimal volume that is sufficient for complete liquid replacement. 

To address this challenge, we utilized COMSOL Multiphysics (version 6.0), a simulation software, to study the minimal liquid volume and optimal flow rate for two types of fluidic chambers in oblique–incidence reflectivity difference (OI-RD) biosensors. OI-RD is a label-free technique that utilizes fluidic chambers to measure biomolecular interactions in solutions [22,23,24,25,26,27,28]. In our study, we examined the effects of flow rate, positions on the substrate surface bearing samples, and depth of the chamber on the required volume for complete fluidic replacement. Our findings indicate that the depth of the fluidic chamber is the primary factor influencing the required liquid volume, with the volume following a quadratic function in relation to depth. Furthermore, we observed that the positions on the substrate surface bearing samples also have an impact on the required fluid volume, while the flow rate does not affect the fluid volume in any significant way.

## 2. Materials and Methods

### 2.1. Geometry of Fluidic Chambers

**Figure 1:**
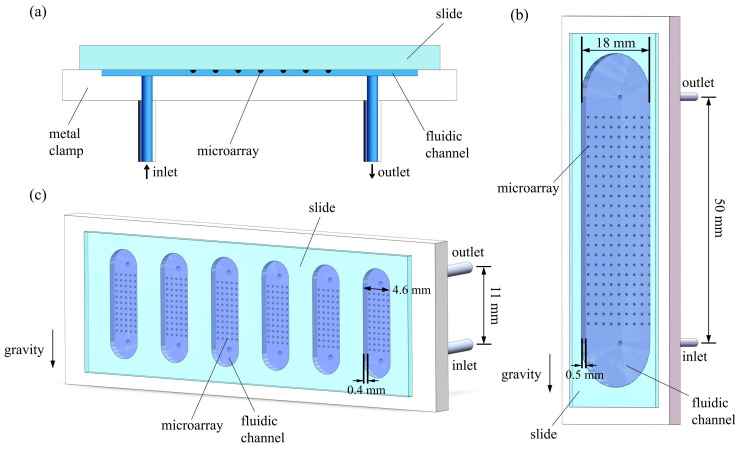
(**a**) Schematic of a flow cell, consisting of a metal clamp, a fluidic channel, and a glass slide with microarrays; (**b**) 3D representation of the flow cell with a large fluidic chamber; (**c**) 3D representation of the flow cell with six small fluidic chambers.

Figure 1a illustrates a flow cell consisting of a metal clamp, a fluidic channel, and a glass slide containing microarrays. The metal clamp securely holds the fluidic channel and the glass slide together, creating a fluidic chamber designed for liquid manipulation. The fluidic chamber includes an inlet and an outlet to facilitate the inflow and outflow of fluids.

OI-RD biosensors employ fluidic chambers of different sizes for various applications. Figure 1b illustrates a 3D representation of a large fluidic chamber, measuring 18 mm in width, 0.5 mm in depth, and with a spacing of 50 mm between the inlet and the outlet. The volume of the fluidic chamber is 576.4 μL. The walls of the fluidic channel in the large chamber are made of PEEK (Polyetheretherketone). This large fluidic chamber is designed to accommodate a glass slide containing tens of thousands of biomolecules and is commonly used for primary screenings.

Figure 1c shows a 3D representation of a flow cell containing six small chambers. Each small chamber measures 4.6 mm in width, 0.4 mm in depth, and has a spacing of 11 mm between the inlet and the outlet. The volume of each small chamber is 26.9 μL. The walls of the fluidic channel in each small chamber are made of titanium. This fluidic chamber is specially designed to accommodate a glass slide with six independent microarrays, making it ideal for secondary screenings and real-time analyses of biomolecular interactions.

### 2.2. Simulation Software and Content of Simulations

To determine the minimal volume of fluids required for complete solution replacement, COMSOL Multiphysics is used to simulate the dynamics of solution replacement within the fluidic chamber. This commercially available finite element method software offers a user-friendly graphical interface that facilitates the construction of numerical models with various geometries and governing equations. COMSOL encompasses various capabilities, including simulations of AC/DC phenomena, chemical reaction engineering, geo-mechanics, heat transfer, RF (radio frequency) phenomena, and fluid dynamics, enabling accurate modeling and analysis of different studies [29,30,31]. For this study, the Computational Fluid Dynamics (CFD) module is utilized, which is specially designed to simulate fluid flow and related processes, providing the necessary tools and capabilities to accurately model and analyze fluid dynamics in this study.

### 2.3. Mesh, Boundary Conditions, and Initial Conditions

The geometric models of two fluidic chambers are created using SOLIDWORKS (version 2021 SP2.0), a 3D design software. The CAD files representing these fluidic chambers are then imported into COMSOL Multiphysics for simulations. Free tetrahedral meshing is applied in all simulations, with user-controlled mesh selected in a sequence type. The element size is calibrated for the fluid dynamics. For the large fluidic chamber, the maximum element size is set to 0.15 mm, and the minimum element size is 0.05 mm. The total number of elements in this mesh is 5.14 × 10^6^. For the small chamber, the maximum element size is reduced to 0.049 mm, and the minimum element size is 0.016 mm. The total number of elements in this mesh is 3.90 × 10^6^. No significant differences are observed with finer meshes for both chambers.

In the CFD module, the simulations utilize the laminar flow interface and the transport of diluted species interface. A no-slip condition is applied to all boundaries of the fluidic chambers. The inlet boundary condition is set as a velocity condition, where the normal inflow velocity is determined by the fluidic flow rate. The outlet is set as a pressure condition at 1.013 × 10^5^ Pa (1 atm). The outlet boundary conditions include compensating for hydrostatic pressure approximation, enforcing normal flow, and suppressing backflow. Gravitational acceleration is applied in the direction indicated in Figure 1b,c.

For the simulations, the protein solution contains 500 nM of bovine serum albumin (BSA) with a diffusion coefficient of 6.07 × 10^−11^ m^2^/s [32]. It is worth noting that the diffusion coefficients of most proteins are generally in the same range (10^−11^ m^2^/s) [32]. Therefore, the specific type of protein used in the simulations do not have a significant impact on the results. The buffer solution is an HEPES buffer. The density and dynamic viscosity of the fluids are set to the same values as water as the physical properties of BSA and the buffer solution closely resemble those of water. The dynamic viscosity of the fluid is temperature-dependent, and for these simulations, the temperature is set to room temperature, which is 293.15 K.

## 3. Results

### 3.1. Simulation for the Large Fluidic Chamber

#### 3.1.1. Distribution of Protein Concentration on Microarray Surface after Flowing Protein at an Equivalent Volume to the Large Fluidic Chamber

**Figure 2:**
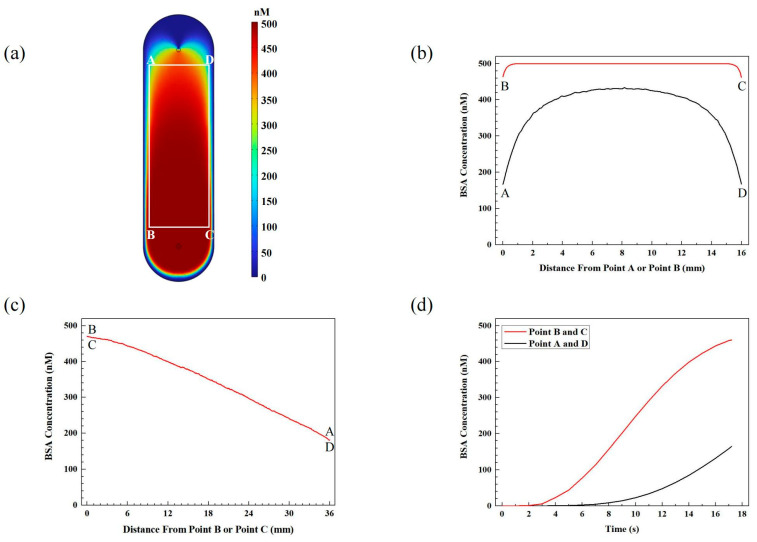
(**a**) Concentration distribution of BSA on the substrate surface after the injection of BSA at an equivalent volume of the large fluidic chamber; (**b**) concentration distribution of BSA along line AD and line BC in (**a**); (**c**) concentration distribution of BSA along line BA and line CD in (**a**); (**d**) the increase in BSA concentration over time at point A, B, C, D in (**a**) during BSA injection.

The concentration distribution of BSA across the sample surface was simulated by flowing 576.4 μL of BSA (equivalent to the volume of the large fluidic chamber) at a concentration of 500 nM from the inlet to the outlet at a flow rate of 2 mL/min. Figure 2a illustrates the concentration distribution of BSA on the top surface of the fluidic chamber, which represents the substrate surface bearing samples. Samples are immobilized as microarrays within the region marked by the rectangle ABCD, which is 36 mm in length and 16 mm in width. Line BC and AD are both 18 mm away from the center of the fluidic chamber, and line AB and CD are both 8 mm away from the center of the fluidic chamber. It is obvious that the BSA concentration across the substrate is non-uniform, and the concentration near the outlet deviates significantly from the original concentration.

Figure 2b shows the concentration distribution of BSA along line BC and line AD. The majority of points along line BC maintain a concentration of 500 nM, which is consistent with the original concentration. However, the points in close proximity to point B or point C deviate from this trend. At a distance of 1.2 mm from point B or point C, the concentration begins to decrease gradually until it reaches a minimum of 460 nM at these points. This minimum concentration corresponds to approximately 92% of the original concentration. On the contrary, the highest concentration of BSA along line AD is observed at the midpoint, with a concentration of approximately 430 nM, which is 86% of the original concentration. Moving away from the midpoint towards point A or point D, the concentration gradually decreases. At point A or point D, the concentration reaches its minimum value of around 167 nM, which is only 33.4% of the original concentration.

Figure 2c depicts the concentration distribution of BSA along line BA and line CD, which overlap with each other. Moving away from point B and C, the concentration gradually decreases from 460 nM to 167 nM along both lines. It is evident that flowing an equivalent volume of BSA solution to the fluidic chamber’s volume cannot guarantee complete replacement of the buffer solution for positions located far away from the inlet. The concentrations at points A and D can be utilized to determine the minimum volume of BSA required for complete replacement of the buffer solution throughout the entire fluidic chamber.

Figure 2d illustrates the dynamic change in BSA concentration over time for points A, B, C, and D during the injection of BSA solution at a flow rate of 2 mL/min. At points B and C, the BSA concentration begins to increase after 3 s of injection and reaches 460 nM after 17.3 s of injection, when an equivalent volume to the large chamber injection is being injected into the fluidic chamber. At points A and D, the BSA concentration begins to increase after 7 s of injection and reaches 167 nM after 17.3 s of injection, when an equivalent volume to the large chamber injection is being injected into the fluidic chamber. The concentration at points B and C increases at a faster rate compared to points A and D, which supports the rationale for studying the concentrations at points A and D to determine the minimal volume of BSA needed for complete replacement of the buffer solution.

#### 3.1.2. Minimal Volumes of Protein Solution for the Large Fluidic Chamber

**Figure 3:**
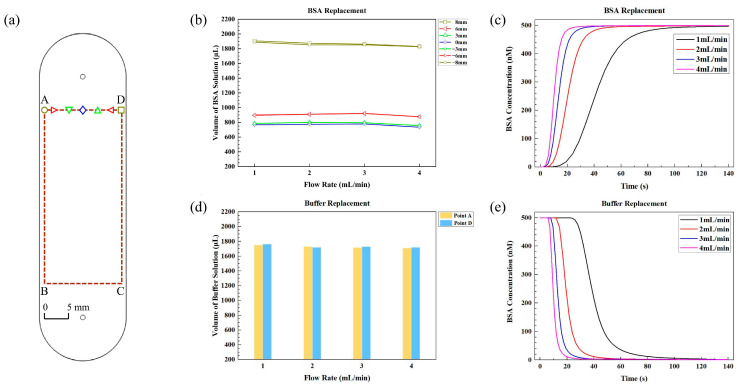
(**a**) The front view of the large fluidic chamber with the microarray area ABCD marked by red dashed lines. The graphic symbols, displayed in different shapes and colors, represent distinct sampling points. These symbols correspond to the legends in (**b**); (**b**) minimal volumes of BSA solution required for different points along line AD to achieve 99% of the original BSA concentration in the large fluidic chamber at various flow rates; (**c**) the changes in BSA concentration over time during BSA replacement at point D in (**a**) at different flow rates; (**d**) minimal volumes of buffer solution required for point A and point D in (**a**) to achieve 1% of the BSA concentration at various flow rates; (**e**) the changes in BSA concentration over time during buffer replacement at point D in (**a**) at different flow rates.

To determine the volume of BSA required for complete replacement of the buffer solution (reaching 99% of the original concentration of BSA), seven distinct points along line AD (Figure 3a) were examined. Figure 3b illustrates the minimal volumes of BSA solution needed at each point for four different flow rates. It is evident that the volume of BSA required for complete replacement of the buffer is dependent on the locations rather than the flow rates. On the central axis of the microarray area, approximately 773.2 μL of BSA solution is required, which is approximately 1.34 times the volume of the large fluidic chamber. For points located 3 mm, 6 mm, and 8 mm away from the central axis, the required volumes of BSA solution for complete replacement are approximately 799.7 μL, 909.2 μL, and 1873.9 μL, respectively. These volumes correspond to approximately 1.39 times, 1.58 times, and 3.25 times the total volume of the chamber. The required volumes for points located −3 mm, −6 mm, and −8 mm away from the central axis are similar to those for points located 3 mm, 6 mm, and 8 mm away from the axis, respectively. Therefore, the minimal volume for complete replacement of buffer by BSA is 1873.9 μL, which is about 3.25 times the total volume of the chamber.

Although the flow rate does not have a significant impact on the required volume of BSA solution, it does affect the time needed to achieve complete protein replacement. Figure 3c illustrates the change in BSA concentration over time at various flow rates for point D in Figure 3a. Clearly, the BSA concentrations change more rapidly at higher flow rates. At flow rates of 1 mL/min, 2 mL/min, 3 mL/min, and 4 mL/min, the time required to reach the desired concentration is 114.4 s, 56.2 s, 37.2 s, and 27.5 s, respectively. A higher flow rate enables faster attainment of the desired concentration without increasing the volume of BSA solution.

#### 3.1.3. Minimal Volumes of Buffer Solution for the Large Fluidic Chamber

The simulation of replacing 500 nM BSA with a buffer solution is also conducted. Since point A and point D determine the minimal volumes required for a complete replacement of fluid, Figure 3d solely illustrates the volumes of buffer solution required to completely replace the BSA solution (reaching 1% of the original BSA concentration) for these two points at different flow rates. Similar to when BSA replaces the buffer, the volume required for complete buffer replacement by BSA also depends on the locations rather than the flow rate.

At a flow rate of 2 mL/min, the volume of buffer solution needed for point D is calculated to be 1719.3 μL, which is approximately 2.98 times the total volume of the large chamber. Additionally, the volume of buffer solution required for point A is similar to that of point D. The similarity in volume requirements between these two points indicates that the volume needed for replacing BSA and the volume needed for buffer replacement are almost identical.

Figure 3e illustrates the change in BSA concentration over time at point D in Figure 3a during the protein replacement process at various flow rates. At flow rates of 1 mL/min, 2 mL/min, 3 mL/min, and 4 mL/min, the time required for complete replacement of BSA is 105.7 s, 51.6 s, 34.6 s, and 25.8 s, respectively. Once again, it is evident that the BSA concentration decreases more rapidly at higher flow rates, enabling faster replacement without the need to increase the volume of the buffer solution.

#### 3.1.4. Minimal Volumes of Protein Solution for Large Fluidic Chamber with Various Depths

**Figure 4:**
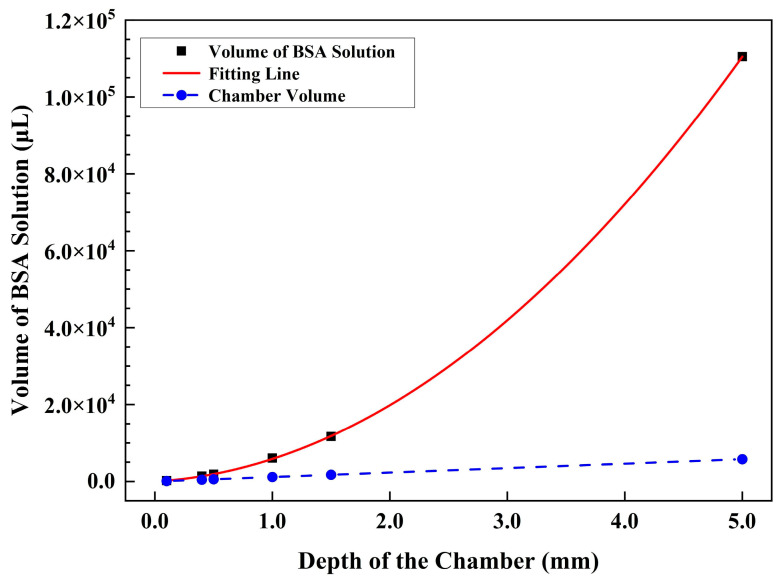
Variation of BSA volume and chamber volume with the depth of the large chamber, along with the fitting curve between BSA volume and chamber depth.

The study also examined the volume needed to completely replace the fluid in the large fluidic chamber at various depths, using a flow rate of 2 mL/min at point D. For depths of 0.1 mm, 0.4 mm, 0.5 mm, 1.0 mm, 1.5 mm and 5.0 mm, the minimal volumes of BSA solution are 225.2 μL, 1366.4 μL, 1873.9 μL, 6072.0 μL, 11,680.0 μL, and 110,458.1 μL, respectively. These volumes correspond to 1.95 times, 2.96 times, 3.25 times, 5.27 times, 6.75 times, and 19.16 times the volume of the respective fluidic chamber at different depths. It is evident from Figure 4 that the volumes increase significantly with depth, and the ratio of BSA volume to chamber volume is not constant.

For a depth of 5.0 mm, the volume ratio is approximately 20 times, whereas the ratio for a point 8 mm away from the central line is less than four times. These findings suggest that the volume of BSA is more influenced by the depth of the chamber than by the position on the substrate bearing samples.

Further analysis revealed that the required volumes of BSA solution exhibit a quadratic function of depth, as follows:

*V* = 4058.71·*d*^2^ + 1797.08·*d* (1)

*V* represents the BSA volume, *d* represents the depth, and the fitting *R*^2^ value is 0.99999. The quadratic relationship between BSA volume and depth highlights the significance of depth as a crucial parameter that influences the liquid volume required for complete fluidic replacement.

### 3.2. Simulation for the Small Fluidic Chamber

#### 3.2.1. Minimal Volumes of Protein Solution for the Small Fluidic Chamber

**Figure 5:**
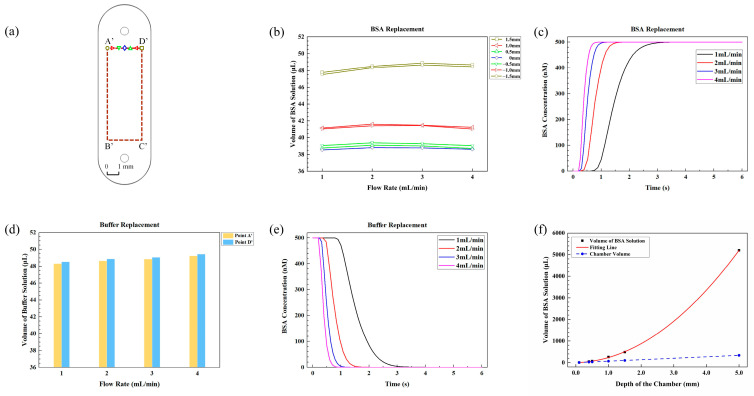
(**a**) Front view of the small fluidic chamber, with the microarray area A’B’C’D’ indicated by red dashed lines. The graphic symbols, represented by various shapes and colors, correspond to the legends in (**b**); (**b**) minimal volumes of BSA solution required for seven distinct points along line A’D’ to achieve 99% of the original BSA concentration in the small fluidic chamber at various flow rates; (**c**) the changes in BSA concentration over time during BSA replacement at point D’ in (**a**) at different flow rates; (**d**) minimal volumes of buffer solution required for point A’ and point D’ in (**a**) to achieve 1% of the BSA concentration at various flow rates; (**e**) the changes in BSA concentration over time during buffer replacement at point D’ in (**a**) at different flow rates; (**f**) variation in BSA volume and chamber volume with the depth of the small chamber, along with the fitting curve between BSA volume and chamber depth.

Figure 5a shows the microarray area, marked by red rectangle A’B’C’D’, with a length of 8 mm and a width of 3 mm for the small fluidic chamber. Figure 5b demonstrates the minimal volume of BSA required for seven points along line A’D’ in Figure 5a at different flow rates. It is evident that the volume of BSA needed for complete replacement of the buffer depends on the locations rather than the flow rates.

At a flow rate of 2 mL/min, 38.8 μL of BSA solution is required on the central axis of the microarray area, which is about 1.44 times the total volume of the small fluidic chamber. For points ± 0.5 mm, ± 1.0 mm, and ± 1.5 mm away from the central axis, the minimal volumes of BSA solution required are 39.1 μL, 41.6 μL, and 48.5 μL, respectively. These amounts correspond to approximately 1.45 times, 1.54 times, and 1.80 times the total small fluidic chamber volume.

Figure 5c demonstrates the change in BSA concentration over time for point D’ at four different flow rates. It is observed that higher flow rates result in faster BSA replacement, leading to a shorter time to reach the final concentration. Specifically, at flow rates of 1 mL/min, 2 mL/min, 3 mL/min, and 4 mL/min, the time required for full protein replacement is 2.87 s, 1.45 s, 0.98 s, and 0.73 s, respectively. Thus, higher flow rates are preferred for liquid transfer to enable faster replacement with the same volume of the BSA solution.

#### 3.2.2. Minimal Volumes of Buffer Solution for the Small Fluidic Chamber

The replacement of 500 nM BSA with buffer solution is also simulated. Figure 5d shows the volumes of buffer solution required to completely replace the BSA solution (reaching 1% of the original BSA concentration) for point A’ and point D’ in Figure 5a at different flow rates. Again, flow rate has little effect on the buffer volume.

At a flow rate of 2 mL/min, the minimal volumes of buffer solution required to fully replace BSA are as follows: for point D’, 48.9 μL of buffer solution is required, which is about 1.82 times the total volume, and the volume of buffer solution required for point A’ is close to that for point D’. Thus, for the small fluidic chamber, 48.9 μL of buffer solution ensures complete replacement of 500 nM BSA.

Figure 5e illustrates the change in BSA concentration over time at point D’ following buffer replacement at different flow rates. As the flow rate increases from 1 mL/min to 2 mL/min, 3 mL/min, and 4 mL/min, the time required for complete replacement decreases from 2.91 s to 1.47 s, 0.98 s, and 0.74 s, respectively. This result indicates that higher flow rates are also preferred for buffer replacement in the small fluidic chamber.

#### 3.2.3. Minimal Volumes of Protein Solution for Small Fluidic Chambers with Various Depths

The dependence of minimal volumes of BSA solution on the depth of the chamber for point D’ is shown in Figure 5f. As the chamber depth increases from 0.1 mm to 0.4 mm, 0.5 mm, 1.0 mm, 1.5 mm and 5.0 mm, the required volumes of BSA solution are approximately 6.3 μL, 48.5 μL, 71.6 μL, 258.2 μL, 485.4 μL and 5197.9 μL, respectively. These volumes correspond to approximately 0.94 times, 1.80 times, 2.13 times, 3.84 times, 4.81 times and 15.47 times the volume of the respective fluidic chamber with different depths. For the small fluidic chamber, the volume of required BSA solution also increases quadratically with the depth of the chamber, as follows:

*V* = 200.84·*d*^2^ + 35.24·*d* (2)

The fitting *R*^2^ value is 0.99995. Similar to the results of the large fluidic chamber, the small fluidic chamber also shows a quadratic relationship between the BSA volume and depth. This further emphasizes the importance of depth as an important parameter that influences the liquid volume required for complete fluidic replacement.

## 4. Discussion

OI-RD is a technique that detects biomolecular interactions without the need for labeling molecules. This method utilizes fluidic chambers to conduct in situ detection. In order to obtain precise results and minimize sample wastage, it is important to establish the optimal volume and flow rate. Simulations were thus conducted to determine the optimal volume of fluid required for complete liquid replacement in the two types of fluidic chambers used in the OI-RD biosensor.

It was observed that the depth of the chamber played a crucial role in influencing the required liquid volume. Both types of fluidic chambers show a quadratic relationship between the required volume and the chamber’s depth. This quadratic relationship may be due to the increasing distance that protein molecules need to diffuse from the bottom of the chamber to the top substrate surface as the chamber’s depth increases. Consequently, it takes more time for the solution on the substrate surface to reach the desired concentration, resulting in a non-linear relationship between the required volume and the chamber’s depth.

The quadratic relationship between the fluid volume and the chamber’s depth indicates that a fluidic chamber with a smaller chamber depth can help reduce sample consumption. However, in the case of the OI-RD biosensor, it is important to take into account the scattering effect that occurs when the laser beam reaches the bottom of the chamber after passing through the substrate and the liquid. If the chamber depth is too small, scattered laser light can cause significant interference with the detected OI-RD signal, resulting in large noise. Therefore, it is crucial to avoid using excessively small chamber depths. Typically, a chamber depth of 0.4 mm or 0.5 mm is considered the optimal choice to balance sample consumption and the noise level caused by scattered laser light.

Furthermore, the fluidic volume required for complete liquid replacement also depends on the positions on the substrate surface bearing samples. Positions closer to the chamber inlet require a smaller volume of liquid for complete fluidic replacement compared to positions farther away from the inlet. When the microarray spots are not enough to cover the entire substrate surface, it is advisable to immobilize the spots in the area close to the inlet. This area requires less volume and less time to reach the desired concentration. Additionally, it was observed that the flow rate has minimal impact on the required volume for both fluidic chambers.

Understanding the minimal volume of sample solution required to replace the buffer solution in fluidic chambers is highly beneficial, especially for larger chambers, as it reduces sample consumption during primary high-throughput drug screening using the OI-RD sensor. In this type of screening, a solution of the target protein is passed over a small molecule microarray (SMM), which is a glass slide with tens of thousands of compounds immobilized on its surface. This allows us to screen the compounds that can interact with the target sample. We used to flow 4 mL of sample solution into the large chamber of the OI-RD sensor to fully replace the buffer solution. However, our simulations have revealed that less than 2 mL of sample is sufficient for a complete fluidic replacement. This means that the volume of sample solution previously consumed during one primary screening is actually enough for two screenings. Consequently, by using 4 mL of sample solution, we can now perform high-throughput screenings of two small molecule microarrays. As a result, this substantial decrease in sample consumption enables us to screen nearly 28,000 compounds using the same quantity of sample solution, effectively doubling the previous capacity of approximately 14,000 compounds.

When considering the speed of liquid replacement, higher flow rates are preferred as they enable faster replacement of the liquid, facilitating the attainment of the desired final concentration more quickly. The rapid replacement of fluid is crucial for accurately measuring binding kinetics in biomolecular interactions using the OI-RD biosensor. During the measurement of association kinetics in biomolecular interactions, the target solution is introduced into the chamber at a high flow rate to rapidly replace the buffer solution. Subsequently, a slow flow rate is applied to maintain a constant target concentration during measurement. It is important for the fluidic replacement to be completed the moment the OI-RD signal begins to rise, which corresponds to the initiation of the biomolecular interaction. Currently, the maximum achievable flow rate for the OI-RD biosensor is 4 mL/min, which is limited by the syringe pump model and syringe diameter. At this flow rate, it takes approximately 0.73 s to completely replace the fluid in the small fluidic chamber, which is sufficient for most binding kinetics experiments. If shorter replacement times are needed for experiments involving very rapid association phases, a syringe with a larger diameter or a location closer to the inlet can be used. This principle also applies to the measurement of the dissociation phase, where a fast flow of buffer is used to fully replace the target solution, followed by a slow flow of buffer to maintain a stable buffer environment.

## 5. Conclusions

The liquid replacement dynamics of two types of fluidic chambers for the OI-RD sensor were simulated using COMSOL Multiphysics. Through these simulations, we have observed that the required volume exhibits a quadratic relationship with the chamber depth, which is a key factor influencing the volume of fluid necessary for complete fluidic replacement. Additionally, we have discovered that the required fluid volume is also dependent on the position on the substrate surface bearing samples, while the flow rate has minimal impact on the required volume. These simulations provide valuable insights for accurate measurements in various applications, including primary high-throughput drug screenings using large fluidic chambers, as well as binding kinetics measurements using small fluidic chambers.

## Data Availability

Data are contained within the article.
